# Novel Syntrophic Populations Dominate an Ammonia-Tolerant Methanogenic Microbiome

**DOI:** 10.1128/mSystems.00092-16

**Published:** 2016-09-13

**Authors:** J. A. Frank, M. Ø. Arntzen, L. Sun, L. H. Hagen, A. C. McHardy, S. J. Horn, V. G. H. Eijsink, A. Schnürer, P. B. Pope

**Affiliations:** a Department of Chemistry, Biotechnology and Food Science, Norwegian University of Life Sciences, Ås, Norway; b Department of Microbiology, Swedish University of Agricultural Science, Uppsala BioCenter, Uppsala, Sweden; c Computational Biology of Infection Research, Helmholtz Centre for Infection Research, Braunschweig, Germany; ExxonMobil Research and Engineering

**Keywords:** anaerobic digestion, biogas, metagenomics, metaproteomics, syntrophic acetate oxidation

## Abstract

Biogas reactors operating with protein-rich substrates have high methane potential and industrial value; however, they are highly susceptible to process failure because of the accumulation of ammonia. High ammonia levels cause a decline in acetate-utilizing methanogens and instead promote the conversion of acetate via a two-step mechanism involving syntrophic acetate oxidation (SAO) to H_2_ and CO_2_, followed by hydrogenotrophic methanogenesis. Despite the key role of syntrophic acetate-oxidizing bacteria (SAOB), only a few culturable representatives have been characterized. Here we show that the microbiome of a commercial, ammonia-tolerant biogas reactor harbors a deeply branched, uncultured phylotype (unFirm_1) accounting for approximately 5% of the 16S rRNA gene inventory and sharing 88% 16S rRNA gene identity with its closest characterized relative. Reconstructed genome and quantitative metaproteomic analyses imply unFirm_1’s metabolic dominance and SAO capabilities, whereby the key enzymes required for acetate oxidation are among the most highly detected in the reactor microbiome. While culturable SAOB were identified in genomic analyses of the reactor, their limited proteomic representation suggests that unFirm_1 plays an important role in channeling acetate toward methane. Notably, unFirm_1-like populations were found in other high-ammonia biogas installations, conjecturing a broader importance for this novel clade of SAOB in anaerobic fermentations.

**IMPORTANCE** The microbial production of methane or “biogas” is an attractive renewable energy technology that can recycle organic waste into biofuel. Biogas reactors operating with protein-rich substrates such as household municipal or agricultural wastes have significant industrial and societal value; however, they are highly unstable and frequently collapse due to the accumulation of ammonia. We report the discovery of a novel uncultured phylotype (unFirm_1) that is highly detectable in metaproteomic data generated from an ammonia-tolerant commercial reactor. Importantly, unFirm_1 is proposed to perform a key metabolic step in biogas microbiomes, whereby it syntrophically oxidizes acetate to hydrogen and carbon dioxide, which methanogens then covert to methane. Only very few culturable syntrophic acetate-oxidizing bacteria have been described, and all were detected at low *in situ* levels compared to unFirm_1. Broader comparisons produced the hypothesis that unFirm_1 is a key mediator toward the successful long-term stable operation of biogas production using protein-rich substrates.

## Abstract (precis)

The microbial production of methane or “biogas” is an attractive renewable energy technology that can recycle organic waste into biofuel. Biogas reactors operating with protein-rich substrates such as household municipal or agricultural wastes have significant industrial and societal value; however, they are highly unstable and frequently collapse due to the accumulation of ammonia. We report the discovery of a novel uncultured phylotype (unFirm_1) that is highly detectable in metaproteomic data generated from an ammonia-tolerant commercial reactor. Importantly, unFirm_1 is proposed to perform a key metabolic step in biogas microbiomes, whereby it syntrophically oxidizes acetate to hydrogen and carbon dioxide, which methanogens then covert to methane. Only very few culturable syntrophic acetate-oxidizing bacteria have been described, and all were detected at low *in situ* levels compared to unFirm_1. Broader comparisons produced the hypothesis that unFirm_1 is a key mediator toward the successful long-term stable operation of biogas production using protein-rich substrates.

## INTRODUCTION

The microbial production of methane, or “biogas,” is an attractive renewable energy technology, as it combines recycling of organic waste with biofuel generation. Efficient and stable production of methane depends on complex microbial interactions that eventually lead to methanogenesis. This last step is accomplished by archaeal members of the community that use one or more of the three methanogenic pathways: the hydrogenotrophic pathway (H_2_ + CO_2_ → CH_4_), the acetoclastic pathway (acetate → CH_4_), and the less common methylotrophic pathway (one-carbon compounds such as methanol → CH_4_). Anaerobic digestion of protein-rich substrates has a distinctly high methane production potential but frequently leads to accumulation of the intermediate metabolite ammonia, which can detrimentally affect the viability and metabolism of individual organisms, as well as the stability of the entire reactor microbiome (1–4). Reports have demonstrated that ammonia levels of >0.08 g/liter disturb the microbiome structure and biogas process, reflected by the accumulation of acetate and reduced methane production (1, 4). Notable changes due to high ammonia levels include the decline of acetoclastic methanogenesis, promoting conversion of acetate via a two-step mechanism involving acetate oxidation to H_2_ and CO_2_ (2, 4, 5), followed by hydrogenotrophic methanogenesis through a syntrophic interaction. Despite their key role in high-ammonia reactors, relatively little is known about syntrophic acetate-oxidizing bacteria (SAOB) and their roles in biogas processes. Very few culturable SAOB have been described, all of which typically are slow growers (6) and have numerically small *in situ* populations (5, 7). Molecular studies using targeted functional gene screens (8), DNA stable carbon isotopic probing (9), and protein-stable isotope probing (10) have suggested important roles for uncultured SAOB (4).

This study sought to gain more insight into SAOB populations that accommodate high-ammonia conditions and to generate a more detailed understanding of the microbiology and biochemistry of anaerobic digestion in general. We have carried out analysis of a commercial biogas reactor in Sweden (here referred to as CD01) that has a long history of productive stable operation under high free-ammonia levels of >0.2 g/liter and up to 0.4 g/liter (5, 11, 12). Initial studies carried out in our labs indicated that the CD01 microbiome produces methane predominantly via syntrophic acetate oxidation (SAO) (5). We present a detailed reconstructed population-genome annotation of a novel and uncultured phylotype, here referred to as unFirm_1. Quantitative metaproteomic analysis suggests that unFirm_1 highly expresses SAO pathways that are key to maintaining the flow of carbon from acetate to methane. The importance of unFirm_1 in comparison to other culturable SAOB and biogas reactors is also explored.

## RESULTS AND DISCUSSION

### Microbiome analysis of the CD01 reactor reveals high metaproteomic detection of the numerically abundant unFirm_1 phylotype.

**FIG 1:**
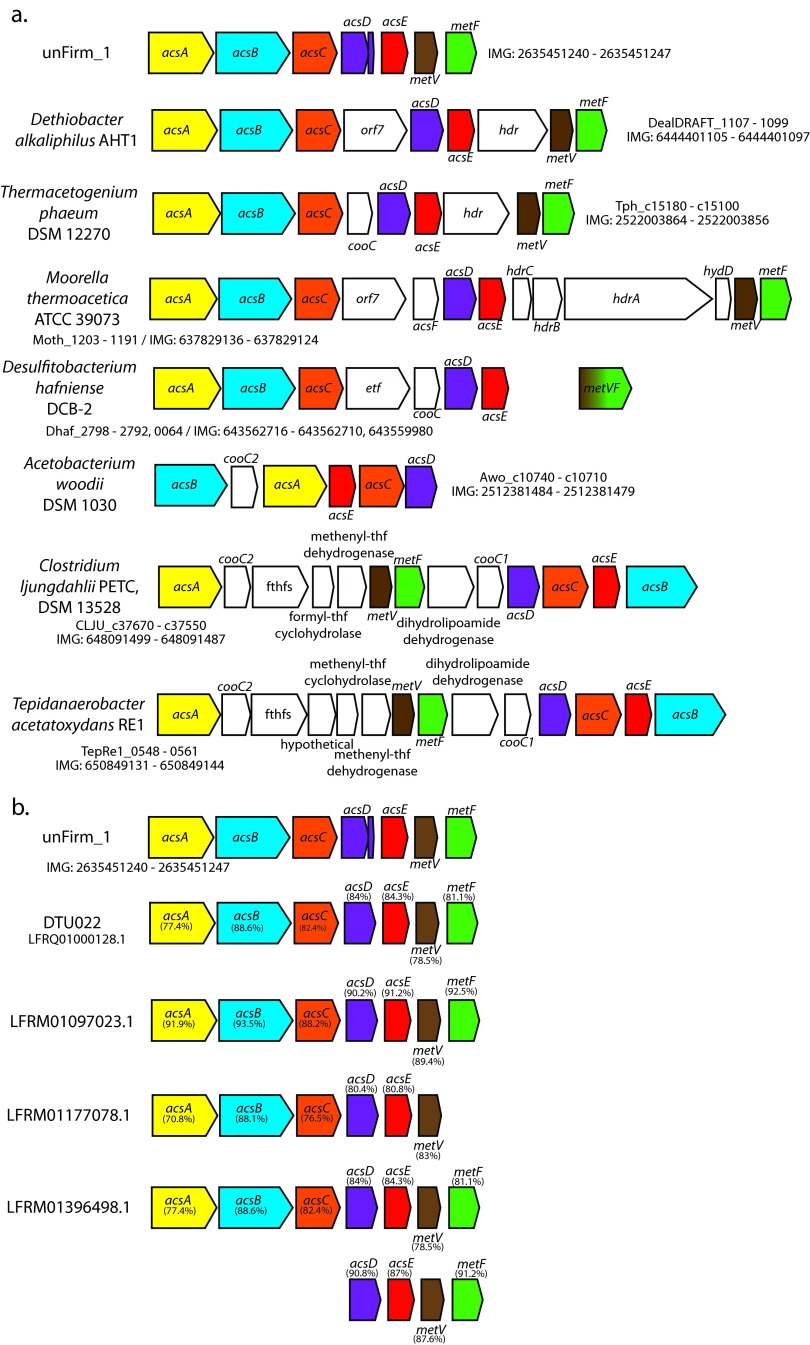
The acetyl-CoA synthase/carbon monoxide dehydrogenase (Acs) operon gene organization of unFirm_1 and comparisons with sequenced acetogen genomes (a) and biogas metagenomic data (b). Values next to the gene names in panel b correspond to the amino acid sequence identities between gene representatives from unFirm_1 and each contig determined by BLASTP. Sequence identifiers from NCBI and, if available, IMG sequence identifiers are provided for each gene/contig. Genes and products: *acsA*, carbon monoxide dehydrogenase/actyl-CoA synthase beta subunit; *acsB*, carbon monoxide dehydrogenase/actyl-CoA synthase alpha subunit; *acsC*, corrinoid/Fe-S protein large subunit; *acsD*, corrinoid/Fe-S protein SSU; *acsE*, methyl-tetrahydrofolate methyltransferase; *metV*, methylene-tetrahydrofolate reductase A; *metF*, methylene-tetrahydrofolate reductase B; *orf7*, ferredoxin; *hdr*, heterodisulfide reductase; *cooC*, carbon monoxide dehydrogenase chaperone; *hydD*, hydrogenase; *etf*, electron transfer avoprotein; fthfs, formyl-tetrahydrofolate synthase.

**FIG 2:**
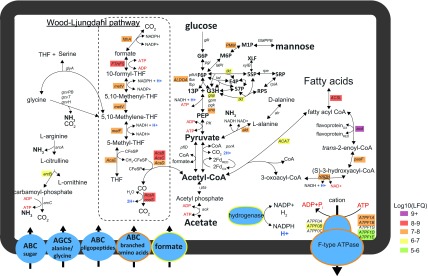
Selected metabolic features of the unFirm_1 phylotype as inferred from genome and proteome comparisons. The unFirm_1 phylotype encoded limited sugar utilization capabilities; however, a complete WL pathway and enzymes inferred to function in fatty acid degradation were observed. Metaproteomic analysis (genes, enzyme complexes, and transport systems are highlighted to indicate quantitative MaxQuant LFQ values) indicated that unFirm_1 was much more detectable in the Link_ADIb sample, with all WL pathway enzymes being detected, as well as genes associated with fatty acid degradation. In particular, the high relative abundance of a unidirectional fructose 1,6-bisphosphate aldolase/phosphatase (ALDOA) supported the hypotheses that unFirm_1 performs gluconeogenesis (anabolic glucose metabolism). Broken lines indicate annotations for which representative genes were not identified in the respective reconstructed genomes. Gene names and identification (IMG gene ID) numbers can be found in Table S1 in the supplemental material. THF, tetrahydrofolate; FTHFS, formyl-tetrahydrofolate synthase; PK, pyruvate kinase; PEP, phosphoenolpyruvate; G6P, glucose-6-phosphate; F6P, fructose-6-phosphate; M6P, mannose-6-phosphate; PMM, phosphomannomutase; ACSL, long-chain-fatty-acid–CoA ligase; HADH, 3-hydroxyacyl-CoA dehydrogenase; GMPPB, mannose-1-phosphate guanylyltransferase; ACAT, acetyl-CoA acetyltransferase; XLF, d-xylulose; G3H, d-glyceraldehyde 3-phosphate; E4P, d-erythrose 4-phosphate; 5SP, d-xylulose 5-phosphate; S7P, sedoheptulose 7-phosphate; 5RP, d-ribulose 5-phosphate; RP5, d-ribose 5-phosphate.

Previous SSU rRNA gene analysis of a sample (Link_ADIa) collected from the high-ammonia/SAO CD01 reactor in Sweden revealed a unique microbiome with several dominant species and an uneven distribution that has seldom been observed in other mesophilic biogas reactors to date (5, 12, 13). Annotation of population genomes that were reconstructed from the Link_ADIa metagenome showed that the second most abundant phylotype (unFirm_1) encoded a carbon monoxide dehydrogenase/acetyl coenzyme A (acetyl-CoA) synthase (Acs) operon, which is characteristic of homoacetogens, including the majority of known SAOB (Fig. 1). The unFirm_1 phylotype was determined to be phylogenetically distinct from its nearest culturable relative, *Pelotomaculum thermopropionicum* (88% small-subunit [SSU] rRNA gene similarity), a syntrophic propionate oxidizer. For unknown reasons, the assembly of unFirm_1-affiliated fragments was problematic (despite its numerical dominance) and required a hybrid HiSeq/PacBio assembly of metagenomic sequence data to generate sufficiently long contiguous fragments (13) (Table 1). In total, a reconstructed partial population-genome representative of unFirm_1 encoded 64 out of 107 conserved single-copy genes (14) and was estimated to be approximately 60% complete (13). Genome annotation was performed, as well as a detailed analysis of metabolic pathways involved in central metabolism and SAO (Fig. 2; see Table S1 in the supplemental material).

10.1128/mSystems.00092-16.4 Table S1  Key metabolic enzymes annotated within the reconstructed genome of unFirm_1. EC numbers (where available), gene names, metaproteomic LFQ values, and IMG gene ID numbers are provided. Download Table S1, DOCX file, 0.03 MB. Copyright © 2016 Frank et al. 2016 Frank et al. This content is distributed under the terms of the Creative Commons Attribution 4.0 International license.

**TABLE 1:** Reactor CD01 characteristics and metagenome and metaproteome features

Characteristic or feature	Link_ADIa metagenome^a^	Link_ADIb metaproteome
Sample features		
Collection yr	2013	2015
Substrate	Slaughterhouse and municipal waste	Municipal waste
Temp (°C)	38	42
pH	7.8	7.7
N-NH_4_^+^ concn (g/liter)	4.6	2.7
NH_3_ concn (g/liter)	0.36	0.22
VFA concn (g/liter)	1.3	0.45
HRT (days)	45	33
OLR (gVS^b^/liter/day)	3.0	4.2
Data set features and genome bins^c^		
Total DNA (assembled) length (Mb)		
*Syntrophomonas* sp.	1.11	
*Tepidanaerobacter* sp.	1.32	
*Thermacetogenium* sp.	0.07	
*Methanosarcina* sp.	0.78	
*Methanoculleus* sp.	2.59	
unFirm_1	2.65^d^	
Total no. of proteins		
*Syntrophomonas* sp.		10
*Tepidanaerobacter* sp.		16
*Thermacetogenium* sp.		0
*Methanosarcina* sp.		8
*Methanoculleus* sp.		30^e^
unFirm_1		176

a Link_ADIa corresponds to the sampling time used for metagenomic characterization of CD01. Genome bins were previously generated by PhylopythiaS+ (the size of each bin is in megabases) (13). Link_ADIb corresponds to the sampling time used for metaproteomic characterization of CD01. The number of proteins associating with each taxonomic group is provided.

b gVS, grams of volatile solids.

c Taxonomic metagenome bins determined by PhyloPythiaS+ analysis on the Link_ADIa metagenome. The total DNA (assembled) length is 189.86 Mb, and the total no. of proteins is 2,312.

d Methodology describing genome reconstruction for unFirm_1 (13).

e Includes proteins affiliated with *Methanoculleus marisnigri* and *M. bourgensis.*

Sampling of the CD01 reactor at a later time point (sample Link_ADIb) revealed a consistent microbiome structure, with unFirm_1 exhibiting similar relative abundance levels (~5%) (see Table S2 in the supplemental material). Furthermore, approximately 78.5% of the Link_ADIb phylotypes were found to correspond to the original Link_ADIa sample. This was despite substrate changes in the commercial operation of the CD01 reactor between the sampling time points to remove slaughterhouse waste and increase the temperature from 38°C to 42 C (Table 1). This change led to a slightly lower ammonia level (0.22 g/liter), which was still above recognized thresholds that are conducive to SAO and hydrogenotrophic methanogenesis (4, 7). Because of the persistent numerical abundance of unFirm_1 over a range of operational conditions, the metaproteome of the CD01 reactor was analyzed to provide functional context for unFirm_1, as well as other known SAOB and methanogens that are important in the carbon flow from the reactor substrate to methane (Table 1). Quantitative metaproteomics (MaxQuant label-free quantification [LFQ]) was used to elucidate relative protein abundance levels, and the two technical replicates analyzed showed 95% protein identification overlap and a Pearson correlation coefficient (*R*) of 0.969 in terms of protein quantification (see Fig. S1 in the supplemental material). The quantitative metaproteomics indicated that many of the more abundant proteins in Link_ADIb map primarily to unFirm_1 (*n* = 176), as well as a hydrogenotrophic methanogen (*Methanoculleus marisnigri*) (see Table S3 in the supplemental material). In total, 2,194 out of the 2,293 identified proteins were mapped to the Link_ADIa metagenome, reaffirming that similar microbial populations exist in both samples (Table 1; see Table S3). The remaining 99 proteins mapped to reference genomes from culturable SAOB and methanogenic representatives that correspond to species commonly found in mesophilic biogas reactors, including those with high ammonia levels (see Table S3).

10.1128/mSystems.00092-16.1 Figure S1  Comparison of the two replicates analyzed by quantitative metaproteomics showing high reproducibility with a Pearson correlation coefficient (*R*) of 0.969. The axes represent log_10_ LFQ values obtained in each replicate, and the colors represent the number of unique peptides associated with each protein. In most cases, the proteins also have several razor peptides associated in addition to the unique peptide(s); see Table S3 for details. Download Figure S1, PDF file, 0.6 MB. Copyright © 2016 Frank et al. 2016 Frank et al. This content is distributed under the terms of the Creative Commons Attribution 4.0 International license.

10.1128/mSystems.00092-16.5 Table S2  Microbiome structure for samples Link_ADIa and Link_ADIb collected from the CD01 reactor. Raw sequence numbers and taxonomy for each OTU are provided for each sample. Approximately 78.5% of the identified OTUs are shared between sampling times, showing that even with a change in reactor substrate and a slight increase in temperature, the community structure was consistent. In both cases, unClos_1 and unFirm_1 are the dominating strains, making up around 40% of the total community at each time point. Download Table S2, XLSX file, 0.03 MB. Copyright © 2016 Frank et al. 2016 Frank et al. This content is distributed under the terms of the Creative Commons Attribution 4.0 International license.

10.1128/mSystems.00092-16.6 Table S3  Proteins from Link_ADIa metagenome-based genome bins, reconstructed genomes of unClos_1 and unFirm_1, and supplementary reference genomes identified by Link_ADIb metaproteomic mapping. The proteins are grouped by species/phylum/genome, and the number of peptides identified, the percentage of sequence covered in each replicate, the protein abundance (log_10_ LFQ) for each replicate, the average abundance and standard deviation, and the IMG annotation are provided. Protein IDs include IMG gene ID numbers (reconstructed genomes), NCBI accession numbers (published genomes), and IMG locus tags for HiSeq (IMG genome ID 3300002977) and PacBio (IMG genome ID 3300006225) metagenomic data sets. Download Table S3, XLSX file, 0.3 MB. Copyright © 2016 Frank et al. 2016 Frank et al. This content is distributed under the terms of the Creative Commons Attribution 4.0 International license.

### Genome and proteome analysis of unFirm_1 suggests metabolic dominance and high SAO activity.

After initial substrate hydrolysis, sugars and amino acids are commonly fermented into acetate, H_2_, and CO_2_, which are the predominant substrates for SAOB and methanogens in a high-ammonia biogas reactor. Recovery of genome and proteome information for unFirm_1 enabled a detailed metabolic reconstruction, which identified a number of key central metabolic pathways essential to acetate oxidation/formation (Fig. 2; see Table S1 in the supplemental material). Genomic analysis identified a number of key pathways, including those inferred in glucose anabolism/catabolism (Embden-Meyerhof-Parnas [EMP] pathway), acetate metabolism (Wood-Ljungdahl [WL] pathway), fatty-acid metabolism, and amino acid metabolism (Fig. 2; see Table S1). Since acetate oxidation is a key stage of biogas production at elevated ammonia levels, particular focus was directed to the WL pathway. This pathway is essential for homoacetogens to perform acetogenesis (acetate produced from CO_2_ and an electron source) and run in reverse by many SAOB that oxidize acetate to CO_2_ and H_2_ (4). A carbon monoxide dehydrogenase/acetyl-CoA synthase (Acs) operon was identified (GeneID 2635451240-47; see Table S1), and the gene products were determined via proteomics to be among the most highly detected, seemingly forming the carbon monoxide dehydrogenase/acetyl-CoA synthase complex and methylene-tetrahydrofolate reductase (MetVF), both key components of the WL pathway (Fig. 2). The genes and organization in the unFirm_1 Acs operon demonstrated similarities to the Acs operon in *Dethiobacter alkaliphilus* AHT1, a non-sulfate-reducing sulfur/thiosulfate-reducing haloalkaliphile isolated from a soda lake (Fig. 1a) (15). In comparison to well-characterized acetogens, the unFirm_1 Acs operon encodes only specific Acs genes, whereas the majority of the others are interspersed with formyltetrahydrofolate synthases, heterodisulfide reductases, carbon monoxide dehydrogenase chaperones, or other genes (Fig. 1a). Additional genes from the WL pathway were also annotated in the unFirm_1 genome and detected at high levels in the metaproteome, including a methylene-tetrahydrofolate dehydrogenase/methenyl-tetrahydrofolate cyclohydrolase, a second MetVF pair, and a formyl-tetrahydrofolate synthase (Fig. 2; see Table S1).

In terms of energy production from autotrophic acetogenesis, it has been demonstrated that no net ATP is generated from the WL pathway. Instead, homoacetogens rely on a chemiosmotic gradient generated by the actions of bifurcating hydrogenases (for regeneration of reducing equivalents), particular types of integral membrane cation pumps, and integral membrane ATP synthases (16). While unFirm_1 encodes representatives of each function type, several instances of dissimilar gene organization and protein subunits were noted compared to other characterized homoacetogens (16) and SAOB (17). For example, the Rnf complex that is used for transporting cations (H^+^ or Na^+^) to generate the chemiosmotic gradient in acetogens appears to be truncated in unFirm_1, having only two subunits (C and D) (GeneID 2635450710-11; see Table S1 in the supplemental material) instead of the typical six. Other acetogens, such as *Moorella thermoacetica*, do not encode the Rnf complex, instead using the energy-conserving hydrogenase (Ech) complex (16). No Ech subunits were found in the unFirm_1 genome, although its incomplete status means that complete Rnf and Ech complexes may have inadvertently been omitted. The RnfC subunit is typically a NADH oxidoreductase, whereas RnfD is part of a multisubunit pump that transports ions out of the cell. Interestingly, in unFirm_1, both of these subunits are encoded adjacent to a formate dehydrogenase (GeneID 2635450712), and both RnfC and the formate dehydrogenase were detected in the metaproteome. Typically, the Rnf complex is not connected to formate dehydrogenases, instead oxidizing ferredoxin and reducing NAD^+^ to generate a cation gradient that is also necessary for ATP generation via the actions of an ATP synthase (16). To determine which cation is pumped by this Rnf complex (either H^+^ or Na^+^), genes corresponding to the F_1_F_0_-type ATP synthase were identified (GeneID 2635451020-27) and the substrate-binding motifs of the C subunit (*atpE*, GeneID 2635451021) were evaluated. As in Na^+^-pumping *Acetobacterium woodii*, there is a Q residue at position 29; however, the ET/ST motif at positions 62 to 64 is absent (see Fig. S2 in the supplemental material), suggesting that the unFirm_1 ATP synthase is H^+^ pumping (18–20). In addition, a second formate dehydrogenase is encoded in close proximity to *nuoE* and *nuoF* NADH ubiquinone oxidoreductase-like subunits and a second *metVF* pair (GeneID 2635450610-14; see Table S1), which were all detected in the unFirm_1 metaproteome (see Table S3). This appears to link the first step of the WL pathway (CO_2_ reduction) to the last (methylene-tetrahydrofolate reduction).

10.1128/mSystems.00092-16.2 Figure S2  Comparison of the F_1_F_0_-type ATP synthase C subunit (AtpE) between H^+^- and Na^+^-translocating representatives from *Thermoanaerobacter kivui* (AIS51827.1), *Acetobacterium woodii* (AFA47026.1), *Propionigenium modestum* (P21905.1), *Bacillus subtilis* (P37815.1), unFirm_1 (2635451021.1), *Synechococcus elongatus* (WP_011243493.1), and *Thermacetogenium phaeum* (AFV12907.1). Highlighted (red) amino acids are those important in dictating which cation is translocated. For Na^+^, the motifs are Q and ET/ST. Download Figure S2, PDF file, 0.5 MB. Copyright © 2016 Frank et al. 2016 Frank et al. This content is distributed under the terms of the Creative Commons Attribution 4.0 International license.

To determine the specific direction of the WL pathway that is employed by unFirm_1 in the CD01 reactor, we investigated three different mechanisms that utilize the WL pathway. This included sugar-fermenting acetogenesis that uses both glycolysis and the WL pathway to produce acetate, autotrophic acetogenesis that uses CO_2_ and H_2_ to produce acetate, and SAO that converts acetate to CO_2_ and H_2_. Closer examination of the EMP pathway revealed several proteins in the proteome, including a fructose 1,6-bisphosphate aldolase/phosphatase (GeneID 2635451940), which indicates that unFirm_1 employs the EMP pathway in an anabolic direction (gluconeogenesis), providing precursors for biosynthesis rather than for catabolism (glycolysis) and ATP generation. Representatives of this enzyme classification are known to have key unidirectional functions in certain homoacetogens/SAOB that perform gluconeogenesis (anabolic glucose metabolism) (see Fig. S3 in the supplemental material). These enzymes have previously been characterized as unidirectional in the homoacetogen *Moorella thermoacetica* (Moth_2266), whereas the closest relative of the unFirm_1 gene was from *Syntrophus aciditrophicus*, a known syntroph (21). Lysine (Lys232) and tyrosine (Tyr348) residues that are essential for aldolase and phosphatase activities, respectively, were present in the unFirm_1 homolog (see Fig. S3 in the supplemental material) (21). These observations suggest that unFirm_1 metabolizes acetate to produce fructose-6-phosphate (possibly toward the pentose phosphate pathway to produce nucleotides and certain amino acids) instead of fermenting sugars to produce acetate as an end product and therefore not acting as a sugar-fermenting acetogen (22). Collectively, the analysis of unFirm_1 in the context of elevated ammonia concentrations that are inhibitory to acetoclastic methanogenesis designates the use of the WL pathway for SAO and reaffirms the phylotype’s role as a highly active SAOB within the CD01 reactor.

10.1128/mSystems.00092-16.3 Figure S3  Comparison of the fructose-1,6-bisphosphate aldolase/phosphatase from *Moorella thermoacetica* (YP_431096.1, Moth_2266) and the putative representative from unFirm_1 with Clustal Omega. The highlighted (red) amino acids are essential for the aldolase (K232) and phosphatase (Y348) activities, respectively. Download Figure S3, PDF file, 0.4 MB. Copyright © 2016 Frank et al. 2016 Frank et al. This content is distributed under the terms of the Creative Commons Attribution 4.0 International license.

### Syntrophic metabolism is prevalent within the CD01 reactor, though culturable SAOB are detected at lower proteomic levels.

A collective evaluation of all of the cultured and uncultured SAOB recovered from the CD01 reactor suggests that acetate oxidation is performed by unFirm_1, as well as close phylogenetic relatives of cultured representatives of *Thermacetogenium phaeum* and *Tepidanaerobacter acetatoxydans* (Table 1; see Table S3 in the supplemental material). Physiological reports on *T. phaeum* and *T. acetatoxydans* have demonstrated both acetogenesis and SAO capabilities in the presence of a hydrogen-consuming methanogen (18, 23). Surprisingly, analysis of these culturable SAOB revealed their limited proteomic detection within the CD01 reactor. Only 16 proteins were identified for a partial *T. acetatoxydans* metagenome bin that was reconstructed from the Link_ADIa sample, whereas only six *T. phaeum* proteins were identified that mapped against a reference genome (see Table S3). No genome or proteome information for other known culturable SAOB (*Syntrophaceticus schinkii* and *Thermotoga lettingae*) was detected. In addition, proteins affiliated with *Syntrophomonas wolfei*, which syntrophically oxidizes larger volatile fatty acids (VFAs) such as butyrate (24), were also detected. Annotation of the detected proteins implies that *S. wolfei* plays an active role in butyrate oxidation, supported by the identification of acyl-CoA dehydrogenases annotated as butyryl-CoA dehydrogenases, an electron transfer flavoprotein beta subunit that works with the butyryl-CoA dehydrogenase, a crotonase, an acetyl-CoA acetyltransferase, a phosphotransacetylase, and an acetate kinase (see Table S3). Similarly, one protein from the *T. phaeum* WL pathway (Acs operon) was detected, implying its SAO capabilities within the CD01 reactor (see Table S3). No *T. acetatoxydans*-affiliated proteins that are associated with SAO were detected in the metaproteome. Fewer proteins of both *S. wolfei* and *T. phaeum* were detected and were at much lower relative-abundance levels than those observed for unFirm_1 (see Table S3). We therefore hypothesize that the novel and abundant unFirm_1 phylotype is an important metabolic contributor to SAO and may be key to understanding the stable operation of biogas reactors at elevated ammonia levels.

**FIG 3:**
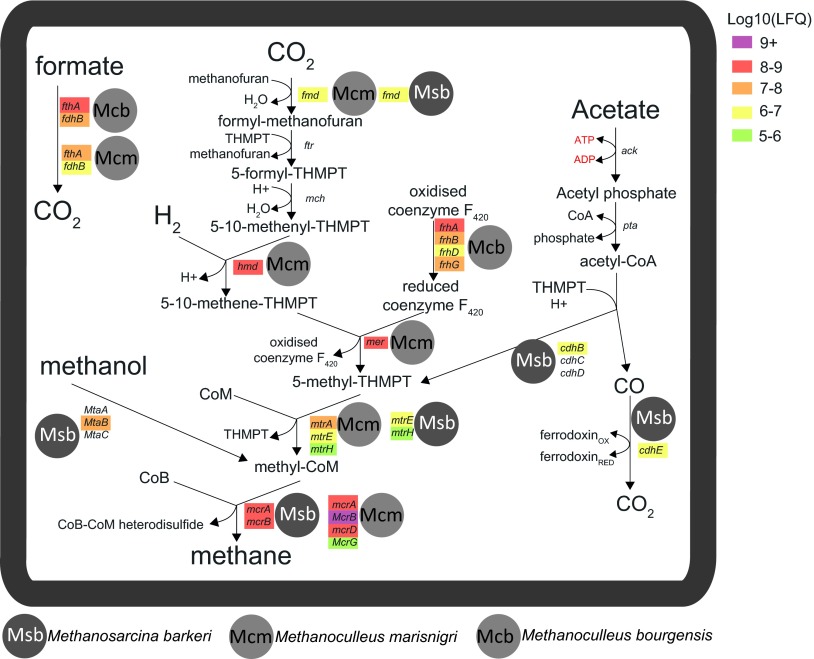
Representation of the major methanogenesis pathways for methanogens detected in high-ammonia biogas reactors. There are three main types of methanogenic pathways: the hydrogenotrophic pathway from H_2_ and CO_2_, the acetoclastic pathway from acetate, and methanogenesis from methylated C_1_ compounds such as methanol. Genes affiliated with archaeal species capable of the three major pathways were detected in the Link_ADIa metagenome. Metaproteomic analysis also detected proteins (genes colored to indicate quantitative MaxQuant LFQ values) from various stages of the three different pathways, with the hydrogenotrophic pathway demonstrating the most highly detected in sample Link_ADIb (see Table S3 in the supplemental material). Genes and products: *mcr*, methyl-coenzyme M reductase; *mta*, methyltransferase; *mtr*, tetrahydromethanopterin *S*-methyltransferase; *mer*, 5,10-methylenetetrahydromethanopterin reductase; *frh*, coenzyme F420 hydrogenase subunit; *hmd*, 5,10-methenyltetrahydromethanopterin hydrogenase; *mch*, methenyltetrahydromethanopterin cyclohydrolase; *ftr*, formylmethanofuran-tetrahydromethanopterin *N*-formyltransferase; *fmd*, formylmethanofuran dehydrogenase; *fdh*, formate dehydrogenase; *cdh*, acetyl-CoA decarbonylase/synthase complex; *pta*, phosphate acetyltransferase; *ack*, acetate kinase; THMPT, tetrahydromethanopterin. Gene identification (IMG gene ID) numbers and peptide information can be found in Table S3.

In addition to syntrophic bacteria, we assessed the methanogens that are present in the CD01 reactor, which detected phylotypes affiliated with *Methanoculleus* sp. and *Methanosarcina* sp. (Table 1). Both of these methanogens are often found in high-ammonia biogas reactors that are metabolically controlled by mesophilic SAO (4, 25). Metabolic reconstruction of the metagenomic binning data and reference genomic information from these genera suggest that methanogenesis in the CD01 reactor can occur via the hydrogenotrophic, acetoclastic, and methanol-derived pathways (Fig. 3). However, proteomic analysis revealed high coverage of the hydrogenotrophic pathway, with associated proteins detected at relative-abundance levels that were among the highest recorded in the Link_ADIb metaproteome (Fig. 3; see Table S3 in the supplemental material). These observations enabled hypotheses that hydrogenotrophic methanogens are predominant in the CD01 reactor, which, when collectively considered with unFirm_1’s metabolism, suggests likely SAO interactions between the two populations. This is supported by previous quantitative PCR experiments that showed high levels of SAOB and hydrogenotrophic methanogens in SAO digesters (5). Interestingly, four CODH/acetyl-CoA synthase subunits were detected in the metaproteome, which suggests that acetate metabolism is also occurring via *Methanosarcina* species, which may include acetoclastic methanogenesis (Fig. 3; see Table S3). In addition, two methanol-5-hydroxybenzimidazyolcobamide methyltransferases from *Methanosarcina* species were detected, indicating that methanol-derived methanogenesis is also possibly occurring.

### Ecological relevance of the unFirm_1 phylotype.

The numerical abundance of and functional influence exerted by unFirm_1 led us to examine the phylotype’s relevance in other ecosystems. Interestingly, unFirm_1 was identified in a controlled biogas study that analyzed bacterial community composition in digesters subjected to high and low ammonia levels (26) (see Tables S4 and S5 in the supplemental material). Closer investigations revealed that unFirm_1 was closely affiliated (99% 16S rRNA gene sequence identity) with uncultured phylotypes that are present only in high-ammonia reactors (SAO3, NH_3_ levels of >0.62 g/liter) and was absent from low-ammonia control reactors (SAO1). This followed trends of other known SAOB, whose relative abundance increased concurrently with ammonia levels (26). Broader SSU rRNA screening (i.e., including environmental sequences) against public data sets in NCBI led to numerous matches of ≥99% (see Table S5), with unFirm_1 being found in 14 different biogas reactor studies involving a wide variety of substrates, including solid waste, pit mud, sludge, landfill soil, cellulose-supplemented manure, and crude oil hydrocarbon-supplemented sludge (see Table S5). However, ammonia was infrequently recorded in many of these other studies, thus limiting our observations and restricting our ability to identify specific correlations of unFirm_1. Comparisons with a large metagenomic data set generated from thermophilic (54°C) laboratory scale reactors revealed only a distant relative of unFirm_1 (93.4% identity) (27). However, closer examinations of the unFirm_1 Acs operon identified identical gene synteny with high amino acid identity (77.4 to 93.5%) in four genomic fragments, including one affiliated with an abundant phylotype (Fig. 1b). Ammonia levels were not recorded in this study; however, the high pH values (>8.0) and high-protein substrate suggest elevated ammonia levels. Collectively, available data indicate that uncultured and hitherto unstudied unFirm_1-like SAOB are detected in various biogas-producing systems, including those that contain high-ammonia conditions.

10.1128/mSystems.00092-16.7 Table S4  Detection of unFirm_1 in bacterial communities originating from controlled biogas digester experiments that contain low and high ammonia levels. Download Table S4, DOCX file, 0.02 MB. Copyright © 2016 Frank et al. 2016 Frank et al. This content is distributed under the terms of the Creative Commons Attribution 4.0 International license.

10.1128/mSystems.00092-16.8 Table S5  Comparison of SSU rRNA gene amplicons for unFirm_1 against the NCBI nr database. Each reported study had one or more matches above 99% sequence identity with the following parameters on NCBI BLASTN: MegaBLAST e = 1e−100, r = 1, q = −1 −F T. Representative sequence accession numbers are reported along with the sample type and associated report, if available. Download Table S5, XLSX file, 0.01 MB. Copyright © 2016 Frank et al. 2016 Frank et al. This content is distributed under the terms of the Creative Commons Attribution 4.0 International license.

### Conclusion.

Ammonia-rich biogas reactors are often associated with elevated levels of VFAs such as acetate, a reduction of acetoclastic methanogenesis, and a syntrophic relationship between SAOB and hydrogenotrophic methanogenesis. From the combined analyses of the CD01 reactor, we have been able to identify a metabolically active novel phylotype that has pathways required for SAO (unFirm_1). Archetypal SAOB were detected in the CD01 reactor, although both their numerical abundance and protein detection levels were significantly lower than those of unFirm_1. Culturable SAOB are typically slow growers, which is considered a disadvantage in the competition for substrates. However, the numerical abundance of the uncultured unFirm_1 phylotype and high coverage and relative detection of proteins inferred in SAO activity suggest it is an important mediator toward the successful long-term stable operation of the CD01 reactor. Proteins from both the WL pathway of unFirm_1 and the hydrogenotrophic methanogenesis pathways of *Methanoculleus* sp. were among the most highly detected in the metaproteome analysis of the CD01 reactor, implying SAO between these populations rather than unFirm_1 acting as a homoacetogen. The identification of unFirm_1-like populations in other reactors from a number of different countries with various operational conditions indicates broader ecological roles for this novel clade of SAOB, for which we present the first genomic characterization and functional interpretation. Isolation efforts targeting unFirm_1, as well as enzyme activity assays, should be pursued to confirm these hypotheses, in concert with strategies that maximize stability and output in high-ammonia systems. Moving forward from individual phylotypes, additional in-depth analyses on a larger scale will be required to holistically characterize the total microbiome of high-ammonia/SAO biogas reactors.

## MATERIALS AND METHODS

### Samples and biogas reactor operational data.

Samples were obtained from a commercial biogas reactor (CD01, located in Sweden) that has endured high ammonia levels for extended periods and has been the focus of other research-and-development activities that have comprehensively described its operational history (5, 11, 12, 28). For the two CD01 samples used in this study, the operating data and substrates used are shown in Table 1. Temperatures, total ammonia nitrogen (TAN) levels, pHs, hydraulic retention times (HRTs), organic loading rates (OLRs), and volatile fatty acid (VFA) levels were obtained from the CD01 reactor operators. These values are based on analytical data from accredited laboratories using standard methods such as Kjeldahl distillation for TAN determination (sum of NH_4_-N [aq] and NH_3_-N [aq]) and high-performance liquid chromatography or Hach-Lange spectrophotometry to analyze VFAs. The fraction of ammonium-nitrogen (NH_4_-N) present as ammonia (NH_3_) was calculated with the formula (29)

$$\frac{\left[{\text{NH}}_{\text{3}}\right]}{\left[{\text{TNH}}_{\text{3}}\right]}={\left(1+\frac{10^{-\text{pH}}}{10^{-\left(0.09018+\frac{2729.92}{T\left(\text{K}\right)}\right)}}\right)}^{-1}$$

where [NH_3_] is the concentration of free ammonia, [TNH_3_] is the total ammonia concentration, and *T*(K) is the temperature (kelvin).

### SSU rRNA genes and metagenomic sequencing.

Total genomic DNA was prepared with the FastDNA Spin kit for Soil (MP Biomedicals, Santa Ana, CA). For SSU rRNA gene sequencing, library preparation was performed in accordance with the manufacturer’s recommendations (Illumina, 2013). V3 and V4 regions of bacterial SSU rRNA genes were amplified with the 341F and 785R primer set (30). The amplicon PCR mixture (25 µl) consisted of 12.5 ng of microbial genomic DNA, 12.5 µl of iProof HF DNA polymerase mix (BioRad), and each primer at 0.2 µM. The PCR was performed with an initial denaturation step of 98°C for 30 s; 25 cycles of denaturation at 98°C for 30 s, annealing at 55°C for 30 s, and extension at 72°C for 30 s; and a final elongation at 72°C for 5 min. A new PCR was carried out to attach unique six-nucleotide indices (Nextera XT Index kit) to the Illumina sequencing adaptors to allow multiplexing of samples. The PCR conditions were as follows: 98°C for 3 min; eight cycles of 95°C for 30 s, 55°C for 30 s, and 72°C for 30°C; and a final elongation step of 72°C for 5 min. AMPure XP beads were used to purify the resulting 16S rRNA amplicons. The 16S rRNA amplicons were quantified (Quant-IT dsDNA HSAssay kit and Qubit fluorometer; Invitrogen, Carlsbad, CA), normalized, and then pooled in equimolar concentrations. The multiplexed library pool was then spiked with 25% PhiX control to improve base calling during sequencing. A final concentration of 8 pM denatured DNA was sequenced on an Illumina MiSeq instrument with the MiSeq reagent v3 kit chemistry with a paired-end, 2 × 300-bp cycle run.

### Metagenomic data sets.

Several shotgun metagenomic data sets with Illumina HiSeq and PacBio circular consensus sequencing reads were previously generated from the Link_ADIa sample and used to develop a protocol for using long reads for assembly and taxonomic binning algorithms (13). In brief, the HiSeq assembly from Link_ADIa resulted in 3.03 million contigs (55,633 above 1 kb) totaling 573.3 Mb, whereas the PacBio assembly generated 40,834 contigs and unassembled reads greater than 1 kb, totaling 60.2 Mb. As previously demonstrated (13), multiple contigs containing SSU rRNA gene fragments and other conserved single-copy genes representative of unFirm_1 were identified in the Link_ADIa HiSeq and PacBio contig data sets. This information enabled the generation of high-quality training data for phylogenomic sequence binning (PhyloPythiaS+) (31) to identify contigs that correspond to unFirm_1, as well as other phylum-, species-, and phylotype-level bins (Table 1). Moreover, hybrid HiSeq/PacBio assembly was used to reconstruct population genomes for unFirm_1 (13) (Table 1). All metagenomic data sets, phylogenomic bins, and reconstructed genomes were functionally annotated and used as reference data sets for the metaproteomic analyses described below.

### SSU rRNA gene-based analyses.

Paired-end reads were joined with the python script join_paired_ends.py (with the default method fastq-join) included in the QIIME v1.8.0 toolkit and quality filtered (at Phred > = Q20) before proceeding with downstream analysis (32). USEARCH61 was used for detection of chimeric sequences, followed by clustering (at 97% sequence similarity) of nonchimera sequences and *de novo* picking of operational taxonomic units (OTUs) (33, 34). Reads were assigned to OTUs with the QIIME v1.8.0 toolkit (32), where uclust (33) was applied to search sequences against a subset of the Greengenes database (35) filtered at 97% identity. Sequences were assigned to OTUs based on their best hit to the Greengenes database, with a cutoff at 97% sequence identity. Taxonomy was assigned to each sequence by accepting the Greengenes taxonomy string of the best-matching Greengenes sequence. Data sets were normalized by using single_rarefaction.py (included in QIIME), and filter_otus_from_otu_table.py was used to filter out OTUs making up less than 0.005% of the total, by using default parameters and –min_count_fraction set to 0.00005, as previously reported (36).

### Functional genomics.

For the identification of protein-coding marker genes, open reading frame (ORF) calling was first performed with the MetaGeneMark (37) version 1 metagenome ORF calling model (gmhmmp -m MetaGeneMark_v1.mod -f G -a -d). The output was subsequently converted into a multiple FASTA file by using the included aa_from_gff.pl script. All unFirm_1 contigs were uploaded to Integrated Microbial Genomes (IMG) Expert Review for functional annotation (38), and overall metabolic pathways were evaluating by using KEGG metabolic maps (39).

The AtpE (ATP synthase, C subunit)-encoding genes from unFirm_1 (2635451021), *Acetobacterium woodii* (AF47026.1), *Thermoanaerobacter kivui* (AIS51827.1), *Synechococcus elongates* (WP_011243493.1), *Bacillus subtilis* (P37815.1), *Thermacetogenium phaeum* (AFV12907.1), and *Propionigenium modestum* (P21905.1) were compared by using Clustal Omega (http://www.ebi.ac.uk/Tools/msa/clustalo/) and default parameters (40, 41). Fructose-1,6-bisphosphate aldolase/phosphatase representatives from unFirm_1 (2635451940) and *Moorella thermoacetica* (Moth_2266, YP_431096) were compared in the same way.

### Metaproteomics.

Proteins were extracted from duplicate Link_ADIb samples by the following method. First, cells and substrate were pelleted at 16,600 × *g* for 3 × 10 min and liquid (secretome) transferred to a new tube (see below). In order to separate cells from substrate, the pellet was dissolved in 1% (vol/vol) methanol–1% (vol/vol) *tert*-butanol–0.1% (vol/vol) Tween 80, (pH 2.0) and the substrate was pelleted by gentle centrifugation at 100 × *g* for 20 s. The cell-containing supernatant was transferred to a new tube, and the substrate pellet was washed again. This was repeated three times to increase the cell count. Cells, now dissociated from the substrate, were finally pelleted by centrifugation at 16,600 × *g* and washed in 10 mM Tris-HCl–1 M NaCl (pH 8.0) prior to cell lysis. Lysis was performed by a bead-beating approach where glass beads (size, ≤106 µm) were added together with lysis buffer (50 mM Tris-HCl, 0.1% [vol/vol] Triton X-100, 200 mM NaCl, 1 mM dithiothreitol) and cells were disrupted by three 60-s cycles with a FastPrep24 (MP Biomedicals, Santa Ana, CA). Debris was removed by centrifugation at 16,600 × *g* for 20 min, and proteins were precipitated by adding ice-cold trichloroacetic acid to a final concentration of 16% (vol/vol) and then incubated at 4°C for 1 h. Proteins were dissolved in SDS sample buffer, separated by SDS-PAGE with a 10% Mini-PROTEAN gel (Bio-Rad Laboratories, Hercules, CA), and then stained with Coomassie brilliant blue R250. The gel was cut into 11 slices, after which proteins were reduced, alkylated, and digested as described previously (42). Prior to mass spectrometry, peptides were desalted with C_18_ ZipTips (Merck Millipore, Darmstadt, Germany) according to the manufacturer’s instructions.

The secretome was concentrated to 500 µl with a centrifugal ultrafiltration filter with a 10-kDa cutoff (Vivaspin; Sartorius, Göttingen, Germany). Proteins were then processed for mass spectrometric analysis while residing in the cutoff filter according to the filter-aided sample prep procedure (43). In brief, denaturing, alkylation, and digestion were accomplished by passing 8 M urea, 50 mM iodoacetamide, and 2 µg of trypsin in Tris-HCl (pH 8.3) through the filter in consecutive steps. Trypsinization was performed overnight on the filter, and peptides were collected the next day by centrifugation (the peptides now pass through the filter). Peptides were desalted with C_18_ ZipTips (Merck Millipore, Darmstadt, Germany) according to the manufacturer’s instructions.

Peptides were analyzed with a nano-liquid chromatography–tandem mass spectrometry (MS/MS) system consisting of a Dionex UltiMate 3000 RSLCnano (Thermo Scientific, Bremen, Germany) connected to a Q-Exactive hybrid quadrupole Orbitrap mass spectrometer (Thermo Scientific, Bremen, Germany) equipped with a nano-electrospray ion source. Samples were loaded onto a trap column (Acclaim PepMap100, C_18_, 5 µm, 100 Å, 300-µm inside diameter [i.d.] by 5 mm; Thermo Scientific) and back flushed onto a 50-cm analytical column (Acclaim PepMap RSLC C_18_, 2 µm, 100 Å, 75-µm i.d.; Thermo Scientific). At the start, the columns were in 96% solution A (0.1% [vol/vol] formic acid), 4% solution B (80% [vol/vol] acetonitrile, 0.1% [vol/vol] formic acid). Peptides were eluted with a 70-min gradient developing from 4 to 13% (vol/vol) solution B in 2 min, 13 to 45% (vol/vol) solution B in 52 min, and finally to 55% solution B in 3 min before the wash phase at 90% solution B, all at a flow rate of 300 nl/min. In order to isolate and fragment the 10 most intense peptide precursor ions at any given time throughout the chromatographic elution, the Q-Exactive mass spectrometer was operated in data-dependent mode to switch automatically between Orbitrap MS and higher-energy collisional dissociation Orbitrap MS/MS acquisition. The selected precursor ions were then excluded for repeated fragmentation for 20 s. The resolution was set to *R* = 70,000 and *R* = 35,000 for MS and MS/MS, respectively. For optimal acquisition of MS/MS spectra, automatic gain control target values were set to 50,000 charges and a maximum injection time of 128 ms.

A total 24 raw MS files (12 × 2 technical replicates) were analyzed by MaxQuant (44) version 1.4.1.2, and proteins were identified and quantified by using the MaxLFQ algorithm (45) to obtain adequate normalization. All proteins from both the secreted and intracellular fractions were pooled for data analysis, and only proteins included in both duplicate sets were used for further analysis. The pooled data were searched against a sample-specific database generated from Link_ADIa metagenomic contigs that were organized into phylogenomic bins by PhyloPythiaS+ (13, 31). In addition to specific metagenomic bins (i.e., unFirm_1), the remaining unassigned Link_ADIa metagenome and reference genome proteins from bacteria and methanogens commonly associated with biogas processes were included: *Methanoculleus bourgensis* (NC_018227.2), *Methanoculleus marisnigri* (NC_009051.1), *Methanosarcina barkeri* (NC_007355.1), *Syntrophomonas wolfei* (NC_008346.1), “*Tepidanaerobacter acetatoxydans*” (NC_019954.2), and *Thermacetogenium phaeum* (NC_018870.1). In total, 275,169 protein sequences were used as a FASTA database for identification by proteomics following the target-decoy strategy. In addition, we added common contaminants such as human keratins, trypsin, and bovine serum albumin, and reversed sequences of all protein entries were concatenated to the database for estimation of false-discovery rates (FDRs). Protein N-terminal acetylation, oxidation of methionine, conversion of glutamine to pyroglutamic acid, and deamination of asparagine and glutamine were used as variable modifications, while carbamidomethylation of cysteine residues was used as a fixed modification. Trypsin was used as a digestion enzyme, and two missed cleavages were allowed. All identifications were filtered in order to achieve a protein FDR of 1%. A minimum of one unique peptide was required for protein quantification.

### Accession numbers.

Data sets are available at the NCBI Sequence Read Archive under BioProject no. PRJNA294734. Complete annotated data for the Link_ADIa HiSeq and PacBio metagenomic data sets can be accessed through the IMG/ER (http://img.jgi.doe.gov/mer/) under IMG genome ID no. 3300002977 and 3300006225, respectively. Similarly, the annotated reconstructed genome of unFirm_1 can be accessed by using IMG genome ID no. 2634166379. The partial 16S rRNA gene for unFirm_1 is available at GenBank (KX553996). The proteomic data have been deposited with the ProteomeXchange consortium (http://proteomecentral.proteomexchange.org) via the PRIDE partner repository (46) with the data set identifier PXD004512.
