# Supplementary material for: Novel Syntrophic Populations Dominate an Ammonia-Tolerant Methanogenic Microbiome
Source: mSystems. 2016 Sep 13;1(5):e00092-16. doi: 10.1128/mSystems.00092-16 (PMC5080403; doi:10.1128/mSystems.00092-16)
Supplement: Table S1 [file sys005162054st4.docx]

**Supplemental Table 1.** Key metabolic enzymes annotated within the reconstructed genome of unFirm_1. E.C. numbers (were available), gene names, metaproteomic label-free quantification (LFQ) values and IMG gene ID numbers are provided.

| Protein/Function | E.C. | gene | IMG gene ID | Proteome (Log10(LFQ)) |
| --- | --- | --- | --- | --- |
| Sugar utilization |  |  |  |  |
| beta-glucosidase | 3.2.1.21 | bgl | 2635452407 | 6.323 |
| mannose-1-phosphate guanylyltransferase / mannose-6-phosphate isomerase | 2.7.7.13/5.3.1.8 | GMPP/MPI | 2635451214 | - |
| phosphomannomutase | 5.4.2.8 | PMM | 2635451213 | 7.339 |
| Glycolysis/Gluconeogenesis |  |  |  |  |
| glucokinase | 2.7.1.2 | glk | 2635451963 | - |
| glucose-6-phosphate isomerase | 5.3.1.9 | pgi | 2635451303 | - |
| 6-phosphofructokinase | 2.7.1.11 | pfkA | 2635453034 | - |
| fructose-bisphosphate aldolase | 4.1.2.13 | fba | 2635451091 | - |
| fructose 1,6-bisphophate aldolase/phosphatase | 3.1.3.11 | ALDOA | 2635451940 | 7.659 |
| triosephosphate isomerase | 5.3.1.1 | tpiA | 2635450822 | 5.953 |
| glyceraldehyde-3-phosphate dehydrogenase | 1.2.1.59 | gap | 2635451304 | 6.811 |
| phosphoglycerate mutase | 5.4.2.12 | gpm | 2635450821 | - |
| phosphoglycerate kinase | 2.7.2.3 | pgk | 2635450824 | - |
| enolase | 4.2.1.11 | eno | 2635452237 | 6.451 |
| enolase | 4.2.1.11 | eno | 2635452132 | 7.650 |
| pyruvate kinase | 2.7.1.40 | pyk | 2635453033 | - |
| pyruvate-ferredoxin/flavodoxin oxidoreductase | 1.2.7.1 | porA | 2635451615 | - |
| pyruvate dehydrogenase E2 component | 2.3.1.12 | DLAT | 2635452255 | - |
| pyruvate formate-lyase | 2.3.1.54 | pflD | 2635451336 | - |
| Pentose phosphate pathway (PPP) |  |  |  |  |
| xylulokinase | 2.7.1.17 | xylB | 2635451984 | - |
| ribulose-phosphate 3-epimerase | 5.1.3.1 | rpe | 2635450899 | - |
| ribose-5-phosphate isomerase | 5.3.1.6 | rpiA | 2635451317 | - |
| transketolase | 2.2.1.1 | tkt | 2635451366 | 6.518 |
| transaldolase | 2.2.1.2 | tal | 2635450864 | 5.531 |
| Fatty acid degradation |  |  |  |  |
| Long-chain-fatty-acid--CoA ligase | 6.2.1.3 | ACSL | 2635450889 | 5.477 |
| Long-chain-fatty-acid--CoA ligase | 6.2.1.3 | ACSL | 2635453202 | 8.540 |
| Long-chain-fatty-acid--CoA ligase | 6.2.1.3 | ACSL | 2635451221 | 7.597 |
| acyl-CoA synthetase |  |  | 2635454626 | - |
| acyl-CoA dehydrogenase | 1.3.8.7 | acd | 2635451685 | - |
| acyl-CoA dehydrogenase | 1.3.8.- | acd | 2635451287 | 5.712 |
| acyl-CoA dehydrogenase | 1.3.8.- | acd | 2635451288 | - |
| acyl-CoA dehydrogenase | 1.3.8.- | acd | 2635451513 | 9.316 |
|  |  |  | 2635451044 | 9.648 |
| Enoyl-CoA hydratase | 4.2.1.17 | paaF | 2635450887 | - |
| Enoyl-CoA hydratase | 4.2.1.- |  | 2635451248 | 7.990 |
| Enoyl-CoA hydratase | 4.2.1.- |  | 2635453233 | - |
| Enoyl-CoA hydratase | 4.2.1.- |  | 2635451220 | 7.614 |
| Enoyl-CoA hydratase | 4.2.1.17 | paaF | 2635451248 | 7.990 |
| 3-hydroxyacyl-CoA dehydrogenase | 1.1.1.35 | HADH | 2635451249 | 7.362 |
| 3-hydroxyacyl-CoA dehydrogenase |  |  | 2635452094 | 7.433 |
| acetyl-CoA acetyltransferase | 2.3.1.9 | ACAT | 2635450965 | - |
| acetyl-CoA acetyltransferase |  | ACAT | 2635451289 | - |
| acetyl-CoA acetyltransferase | 2.3.1.9 | ACAT | 2635451363 | 6.858 |
| Amino acid metabolism |  |  |  |  |
| L- leucine degradation |  |  |  |  |
| leucine dehydrogenase | 1.4.1.9 | leu | 2635452286 | - |
| dihydrolipoamide dehydrogenase | 1.8.1.4 | DLD | 2635452256 | - |
| L-arginine degradation |  |  |  |  |
| arginine deiminase | 3.5.3.6 | arcA | 2635452099 | - |
| ornithine carbamoyltransferase | 2.1.3.3 | arcB | 2635450606 | 6.508 |
| carbamate kinase | 2.7.2.2 | arcC | 2635452101 | - |
| Amino acid transporter |  |  | 2635452098 | - |
| ornithine carbamoyltransferase | 2.1.3.3 | arcB | 2635451597 | - |
| Glycine cleavage system |  |  |  |  |
| glycine hydroxymethyltransferase | 2.1.2.1 | glyA | 2635451316 | - |
| glycine dehydrogenase | 1.4.4.2 | gcvPB | 2635453194 | - |
| aminomethyltransferase | 2.1.2.10 | gcvT | 2635453196 | - |
| glycine cleavage system H protein |  | gcvH | 2635453195 | - |
| dihydrolipoamide dehydrogenase | 1.8.1.4 | DLD | 2635452256 | - |
| Alanine-pyruvate |  |  |  |  |
| alanine racemase | 5.1.1.1 | alr | 2635454587 | - |
| Alanine dehydrogenase | 1.4.1.1 | ald | 2635452360 | 7.281 |
| Wood-Ljungdahl pathway/acetogenesis |  |  |  |  |
| carbon-monoxide dehydrogenase | 1.2.7.4 | acsA | 2635451240 | 8.413 |
| acetyl-CoA synthase | 2.3.1.169 | acsB1 | 2635451241 | 8.608 |
| acetyl-CoA synthase complex subunit gamma | 2.1.1.245 | acsC | 2635451242 | 8.664 |
| acetyl-CoA synthase complex subunit delta | 2.1.1.245 | acsD | 2635451243 | 7.289 |
| 5-methyltetrahydrofolate corrinoid/iron sulfur protein methyltransferase | 2.1.1.258 | acsE | 2635451245 | 7.857 |
| methylenetetrahydrofolate reductase | 1.5.1.20 | metV | 2635451246 | 7.145 |
| methylenetetrahydrofolate reductase | 1.5.1.20 | metF | 2635451247 | 7.907 |
| methylenetetrahydrofolate dehydrogenase / methenyltetrahydrofolate cyclohydrolase | 1.5.1.5 | folD | 2635451694 | 7.855 |
| formiminotetrahydrofolate cyclodeaminase |  |  | 2635451695 | 7.680 |
| formate-tetrahydrofolate ligase | 6.3.4.3 | FTHFS | 2635453168 | 8.606 |
| formate-tetrahydrofolate ligase | 6.3.4.3 | FTHFS | 2635453169 | 8.326 |
| methylenetetrahydrofolate reductase | 1.5.1.20 | metF | 2635450614 | 5.727 |
| methylenetetrahydrofolate reductase | 1.5.1.20 | metV | 2635450613 | - |
| NADH-quinone oxidoreductase subunit F | 1.6.5.3 | nuoF | 2635450612 | - |
| NADH-quinone oxidoreductase subunit E | 1.6.5.3 | nuoE | 2635450611 | - |
| formate dehydrogenase alpha subunit | 1.2.1.43 | fdhA | 2635450610 | - |
| Oxidative phosphorylation/Energy metabolism |  |  |  |  |
| pyrophosphatase | 3.6.1.1 | ppa | 2635451672 | 6.691 |
| NADH-quinone oxidoreductase subunit E | 1.6.5.3 | nuoE | 2635451989 | - |
| NADH-quinone oxidoreductase subunit F | 1.6.5.3 | nuoF | 2635451988 | - |
| NADH-quinone oxidoreductase subunit G | 1.6.5.3 | nuoG | 2635451987 | - |
| Iron only hydrogenase |  |  | 2635452013 | 6.056 |
| formate dehydrogenase beta subunit | 1.2.1.43 |  | 2635452014 | 7.525 |
| NADP-reducing hydrogenase | 1.12.1.3 | hndC | 2635454259 | - |
| formate dehydrogenase iron-sulfur subunit |  | fdoH | 2635450712 | 7.360 |
| electron transport complex protein RnfD |  | rnfD | 2635450711 | - |
| electron transport complex protein RnfC |  | rnfC | 2635450710 | 8.213 |
| ATP synthase F1 subcomplex alpha subunit | 3.6.3.14 | ATPF1A | 2635451024 | 7.255 |
| ATP synthase F1 subcomplex beta subunit | 3.6.3.14 | ATPF1B | 2635451026 | 7.550 |
| ATP synthase F1 subcomplex gamma subunit |  | ATPF1G | 2635451025 | - |
| ATP synthase F1 subcomplex delta subunit |  | ATPF1D | 2635451023 | 5.240 |
| ATP synthase F1 subcomplex epsilon subunit |  | ATPF1E | 2635451027 | 5.392 |
| ATP synthase F0 subcomplex A subunit |  | ATPF0A | 2635451020 | - |
| ATP synthase F0 subcomplex B subunit |  | ATPF0B | 2635451022 | 6.605 |
| ATP synthase F0 subcomplex C subunit |  | ATPF0C | 2635451021 | - |
| Transporters |  |  |  |  |
| oligopeptide transport system substrate-binding protein | - | OppA | 2635453118 | - |
| oligopeptide transport system permease protein | - | OppC | 2635453117 | - |
| oligopeptide transport system ATP-binding protein | - | OppD | 2635453116 | - |
| oligopeptide transport system ATP-binding protein | - | OppF | 2635453115 | - |
| branched-chain amino acid transport system substrate-binding protein | - | LivK | 2635450639 | 7.746 |
| amino acid/amide ABC transporter membrane protein 1, HAAT family (TC 3.A.1.4.-) | - | LivH | 2635450640 | - |
| branched-chain amino acid transport system permease protein | - | LivM | 2635450641 | - |
| branched-chain amino acid transport system permease protein | - | LivG | 2635450642 | - |
| branched-chain amino acid transport system ATP-binding protein | - | LivF | 2635450643 | - |
| sugar transport system substrate-binding protein | TC:3.A.1 | chvE | 2635453093 | - |
| sugar transport system ATP-binding protein | 3.6.3.17 |  | 2635453094 | - |
| sugar transport system permease protein |  |  | 2635453095 | - |
| alanine or glycine:cation symporter, AGCS family |  |  | 2635451993 | - |
| formate transporter | TC:2.A.44 | - | 2635451505 | 6.588 |
