# Supplementary material for: Novel Syntrophic Populations Dominate an Ammonia-Tolerant Methanogenic Microbiome
Source: mSystems. 2016 Sep 13;1(5):e00092-16. doi: 10.1128/mSystems.00092-16 (PMC5080403; doi:10.1128/mSystems.00092-16)
Supplement: Figure S1 [file sys005162054sf1.pdf]

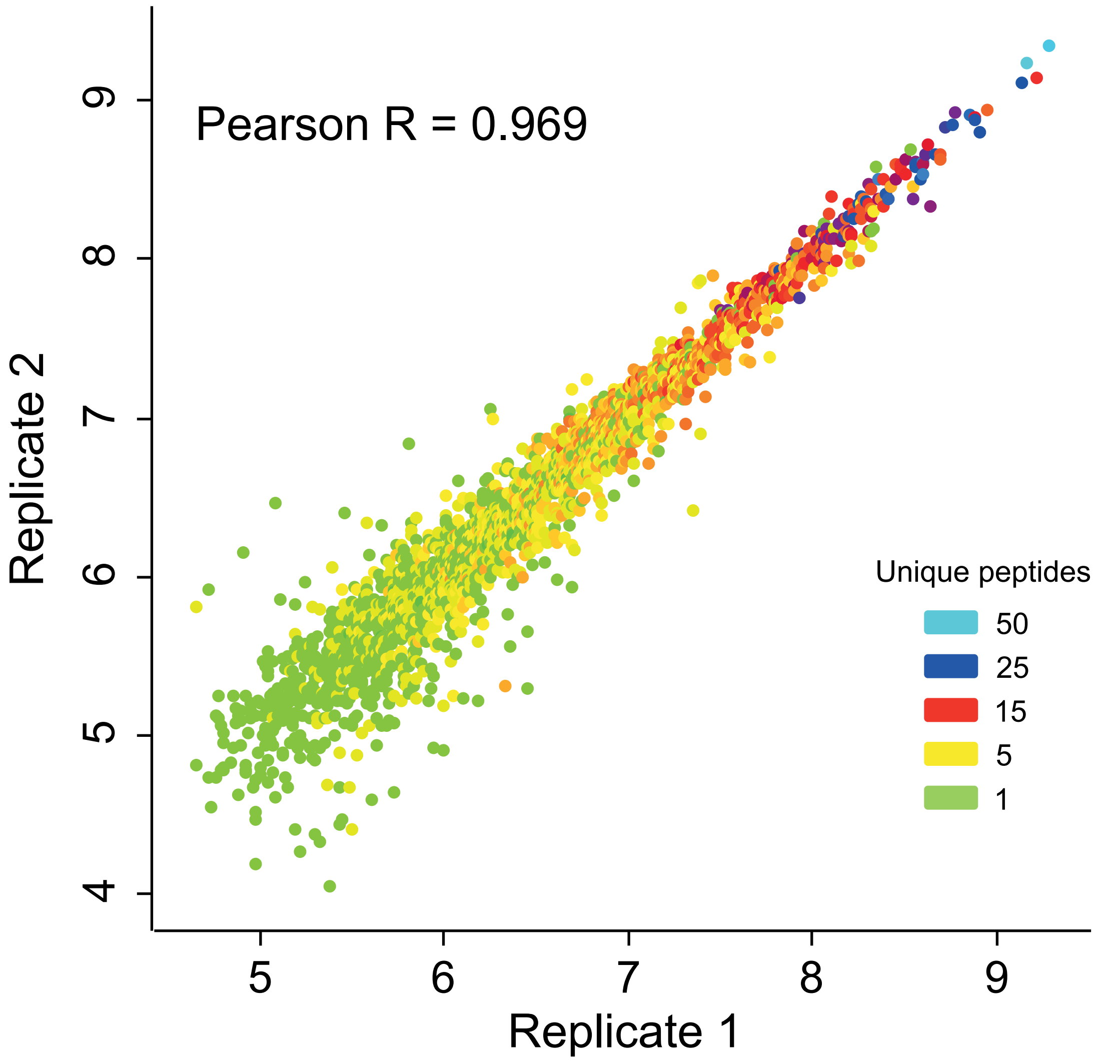

Supplemental Figure 1. Comparison of the two replicates analysed by quantitative metaproteomics showing high reproducibility with Pearson correlation R=0.969. The axes represents log10(LFQ) values obtained in each replicate and the colour represent the number of unique peptides associated with each protein. In most cases, the proteins also have several razor peptides associated in addition to the unique peptide(s), see Table S3 for details.
