# Supplementary material for: Novel Syntrophic Populations Dominate an Ammonia-Tolerant Methanogenic Microbiome
Source: mSystems. 2016 Sep 13;1(5):e00092-16. doi: 10.1128/mSystems.00092-16 (PMC5080403; doi:10.1128/mSystems.00092-16)
Supplement: Figure S2 [file sys005162054sf2.pdf]

```
T.kivui         -----------MDLLAIGAAIA-ALTGIGAGIGIGLATGKAVEAVSRQPEAGGRIMQLLL    48
A.woodii        ----MEGLDFIKACSAIGAGIA-MIAGVGPGIGQGFAAGKGAEAVGRQPEAQSDIIRTML    55
P.modestum      -MDMVLAKTVVLAASAVGAGAA-MIAGIGPGVGQGYAAGKAVESVARQPEAKGDIISTMV    58
B.subtilis      -------------MNLIAAAIAIGLGALGAGIGNGLIVSRTVEGIARQPEAGKELRTLMF    47
unFirm_1        -----------MGLAYVGVALAVALAAFGSALGQGMASRAAMEGTARQPEAAGDIRTTLI    49
S.elongatus     MDSLT------SAASVLAAALAVGLAAIGPGIGQGSAAGQAVEGIARQPEAEGKIRGTLL    54
T.phaeum        -MDLT------SAIIALAVALVMGIATIGPALGQGTAAAKALEGMSRQPEMSGELRTTLI    53
                                :... .  :  .* .:* *       *. .****    :   :.

T.kivui         LGGALAEATAIYGLLVSIMLIIFKP------    73
A.woodii        LGAAVAETTGIYGLIVALILLFANPFF----    82
P.modestum      LGQAIAESTGIYSLVIALILLYANPFVGLLG    89
B.subtilis      MGIALVEALPIIAVVIAFLAFFG--------    70
unFirm_1        LALAFIEALTLFSFVIAILLWTTLPS-----    75
S.elongatus     LSLAFMEALTIYGLVVALVLLFANPFA----    81
T.phaeum        IAMAFMEALTIYGLLIAFLLLGKMG------    78
                :. *. *:  : ..:::::
```

Supplemental Figure 2. Comparison of the F1 F0-type ATP synthase C-subunit (AtpE) between H+ and Na+ translocating representatives from *Thermoanaerobacter kivui* (*T.kiuvi*, AIS51827.1), *Acetobacterium woodii* (*A.woodii*, AFA47026.1), *Propionigenium modestum* (P.modestum, P21905.1), *Bacillus subtilis* (*B.subtilis*, P37815.1), unFirm_1 (2635451021.1), *Synechococcus elongatus* (*S.elongatus*, WP_011243493.1), and *Thermacetogenium phaeum* (*T.phaeum*, AFV12907.1). Highlighted (red) amino acids are those important in dictating which cation is translocated. For Na+, the motifs are Q and ET/ST.
