# Supplementary material for: Novel Syntrophic Populations Dominate an Ammonia-Tolerant Methanogenic Microbiome
Source: mSystems. 2016 Sep 13;1(5):e00092-16. doi: 10.1128/mSystems.00092-16 (PMC5080403; doi:10.1128/mSystems.00092-16)
Supplement: Figure S3 [file sys005162054sf3.pdf]

```
M.thermoacetica      MGERITLSVIKADIGGFVGHSSVHPRLLETAERYLA--ESKLLIDYRVAHVGDDIDLIMT         58
unFirm_1             --LKITLSVIKADIGSIGGHLRPSKTLMKTVEEHIEREKGSLIIDYRISSTGDDIAILFS         58
                        :***********.: **      *::*.*.::   :..*:****:: .**** ::::

M.thermoacetica      HKYGVDCPEIHHLAWNVFLQCTEVARELKLYGAGQDLLSDAFSGNVKGMGPGVAEMEFEE         118
unFirm_1             HREGTGNSAIHELAWNAFVAGTAKAKDEGLYGAGQDLLKDSFSGNIKGLGPAVAEMEFEE         118
                     *: *.    **.****.*:  *  *::  *********.*:****:**:**.********

M.thermoacetica      RKSEPIIVFAADKTEPGAWNLPLYKMFADPFNTIGLVIDPKMHQGFRFEVYDLIK---NE         175
unFirm_1             RENEPFIFFAADKTDPGAYNLPLYLSFADPMHCSGLILSPKMSKGFKYVILDVEHTEKDR         178
                     *:.**:*.******:***:*****  ****:.  **::.*** :**:: : *: :   :.

M.thermoacetica      RVEFSLPEELYDLLVFIGAPGRYCIKSVYSKTTGEIAAVSSTQRLNLMAGRYVGKDDPVC         235
unFirm_1             SIELNAPEDLYDIAALLRDNERFVVSKIYSRETGEQAVAASTTRLHNIAGKYIGKDDPVM         238
                      :*:. **:***: .::    *: :..:**: *** *..:** **. :**:*:******

M.thermoacetica      IVRCQSGLPAVGEALEPFANPHLVAGWMRGSHIGPLMPVGLDQSAPTRFDGPPRVVAMGF         295
unFirm_1             LIRVQGSFPAAGEILSPYSIGHYVAGFMRGSHTGPLMPVQAGC-GTSFFDGPPIVTATAF         297
                     ::* *..:**.** *.*::  * ***:***** ******     . : ***** *.* .*

M.thermoacetica      QLSGGRLVGPQDFFGDVAFDKARQTANEIASYLRSLGPFEPHRLPLEDMEYTTMPEVMAK         355
unFirm_1             CVKNGKFTEPVDCFEHPYWDYVRKKVARKATELRRQGFSGAAMLPYSELEYGGIVEKMKQ         357
                      :. *::. * * * .  :* .*:.. . *: **  *      ** .::**  : * * :

M.thermoacetica      LKNRFVKVDHRDDREPAAEELGAK               379
unFirm_1             LERRFKSEK---------------               366
                     *:.** . .
```

Supplemental Figure 3. Comparison between the fructose-1,6-bisphosphate aldolase/phosphatase from *Moorella thermoacetica* (YP_431096.1, Moth_2266) and the putative representative from unFirm_1 using Clustal Omega. The highlighted (red) amino acids are essential for the aldolase (K232) and phosphatase (Y348) activities, respectively.
