# Supplementary material for: Novel Syntrophic Populations Dominate an Ammonia-Tolerant Methanogenic Microbiome
Source: mSystems. 2016 Sep 13;1(5):e00092-16. doi: 10.1128/mSystems.00092-16 (PMC5080403; doi:10.1128/mSystems.00092-16)
Supplement: Table S4 [file sys005162054st7.docx]

| Digesters | Main Substrate | Temp (ºC) | TAN^a^ (g/L) | NH3^b^ (g/L) | pH | unFirm_1 detection |
| --- | --- | --- | --- | --- | --- | --- |
| SAO1^a^ Day 70-642 | Household waste, egg albumin | 37 | 0.65-0.90 | 0.02 | 7.2-7.4 | No |
| SAO3^a^ Day 70 | Household waste, egg albumin | 37 | 0.82 | 0.02 | 7.2 | No |
| SAO3^a^ Day 141 | Household waste, egg albumin | 37 | 1.9 | 0.09 | 7.6 | No |
| SAO3^a^ Day 225 | Household waste, egg albumin | 37 | 3.3 | 0.30 | 7.9 | No |
| SAO3^a^ Day 442 | Household waste, egg albumin | 37 | 5.5 | 0.62 | 8.0 | Yes |
| SAO3^a^ Day 642 | Household waste, egg albumin | 37 | 6.9 | 0.96 | 7.9 | Yes |

**Supplemental Table 4. Detection of unFirm_1 in bacterial communities originating from controlled biogas digester experiments that contain low‑ and high‑ammonia levels.**

^a^SSU rRNA screens for unFirm_1 were performed against biogas digesters originating from Müller et al (1), matches greater than 99% ID are recorded as positive; ^b^ Total ammonium nitrogen; ^c^ Free ammonia

1. **Müller B, Sun L, Westerholm M, Schnürer A.** 2016. Bacterial community composition and fhs profiles of low- and high-ammonia biogas digesters reveal novel syntrophic acetate-oxidising bacteria. Biotechnol Biofuels **9:**48.
